# Potential Relationship between Inadequate Response to DNA Damage and Development of Myelodysplastic Syndrome

**DOI:** 10.3390/ijms16010966

**Published:** 2015-01-05

**Authors:** Ting Zhou, Peishuai Chen, Jian Gu, Alexander J. R. Bishop, Linda M. Scott, Paul Hasty, Vivienne I. Rebel

**Affiliations:** 1 Greehey Children’s Cancer Research Center, University of Texas Health Science Center San Antonio (UTHSCSA), 8403 Floyd Curl Drive, San Antonio, TX 78229, USA; E-Mails: zhoubaipi@gmail.com (T.Z.); ChenP3@uthscsa.edu (P.C.); bishopa@uthscsa.edu (A.J.R.B.); 2 Department of Cellular and Structural Biology, UTHSCSA, 7703 Floyd Curl Drive, San Antonio, TX 78229, USA; 3 Department of Hematology, Northern Jiangsu People’s Hospital, Yangzhou 225001, China; E-Mail: gujianyz@sina.com; 4 The Cancer Therapy Research Center, UTHSCSA, 7979 Wurzbach Road, San Antonio, TX 78229, USA; E-Mail: hastye@uthscsa.edu; 5 Barshop Institute for Longevity and Aging Studies, UTHSCSA, Texas Research Park Campus, 15355 Lambda Drive, San Antonio, TX 78245, USA; 6 The University of Queensland Diamantina Institute, Translational Research Institute, 37 Kent Street, Woolloongabba, QLD 4102, Australia; E-Mail: l.scott3@uq.edu.au; 7 Department of Molecular Medicine, UTHSCSA, 7703 Floyd Curl Drive, San Antonio, TX 78229, USA

**Keywords:** myelodysplastic syndrome, hematopoietic stem cells, DNA damage response/repair, aging

## Abstract

Hematopoietic stem cells (HSCs) are responsible for the continuous regeneration of all types of blood cells, including themselves. To ensure the functional and genomic integrity of blood tissue, a network of regulatory pathways tightly controls the proliferative status of HSCs. Nevertheless, normal HSC aging is associated with a noticeable decline in regenerative potential and possible changes in other functions. Myelodysplastic syndrome (MDS) is an age-associated hematopoietic malignancy, characterized by abnormal blood cell maturation and a high propensity for leukemic transformation. It is furthermore thought to originate in a HSC and to be associated with the accrual of multiple genetic and epigenetic aberrations. This raises the question whether MDS is, in part, related to an inability to adequately cope with DNA damage. Here we discuss the various components of the cellular response to DNA damage. For each component, we evaluate related studies that may shed light on a potential relationship between MDS development and aberrant DNA damage response/repair.

## 1. Introduction

Hematopoietic stem cells (HSCs) are responsible for maintaining tissue homeostasis during the lifespan of an organism by generating both new stem cells and progeny that will differentiate into myeloid cells (including leukocytes, erythrocytes and platelets) and lymphoid cells (B and T cells). The HSC compartment is heterogeneous, representing roughly three types of stem cells: those that mostly generate myeloid cells, those that generate mostly lymphoid cells and a third type that generates both lineages somewhat equally. Interestingly, the lifespan of these different HSC types vary, resulting in the disappearance of the latter two HSC types with age [1,2]. Although the reason for the difference in survival of these various HSC types is not clear, it fits with early observations that as we age the relative proportion of myeloid cells increases while that of the lymphoid lineage declines [3,4,5]. A comparative gene expression study of young* vs.* old HSCs in mice mirrors this change in myeloid* vs.* lymphoid cells [4]. Other age-related functional changes in the HSC compartment include a decline in regenerative capacity,* i.e.*, the number of cells (including new stem cells) produced by an individual HSC is lower in old HSCs compared to young HSCs [5,6,7,8,9,10,11,12,13].

Stem and progenitor cells isolated from older humans and mice show more H2AX (H2A histone family, member X) staining, thought to be indicative of DNA damage, than those isolated from younger individuals [14,15,16]. These observations, together with comparative gene expression analysis studies in mice, showing that certain DNA repair genes are significantly decreased in old* vs.* young HSCs [17], suggest that DNA repair functions in HSCs also decline with age. However, two recent studies question this notion [14,18]. Flach *et al.* showed that H2AX accumulation in old murine HSCs is not associated with DNA damage, but rather with decreased ribosomal biogenesis and possibly gene silencing. However, the same study also showed that old HSCs suffer significantly from replication stress [14], which, if not dealt with appropriately, can lead to permanent DNA damage (reviewed in [19]). It was found that when old HSCs enter the cell cycle, a significantly larger number of chromosomal breaks were detectable in comparison to young HSCs at the same stage, causing them to stay longer in S-phase, presumably for necessary DNA repair. Beerman *et al.* demonstrated that, indeed, DNA repair pathways are significantly up-regulated in old HSCs as early as 12 h after they enter the cell cycle [18]. Interestingly, this study and the one from Beerman *et al.* seem to suggest that age does not affect DNA repair in HSCs, since no significant increases in DNA breaks or gross chromosomal abnormalities could be detected in old HSCs (compared to young) after completion of the cell cycle [14,18]. However, the absence of DNA breaks [18] does not provide any information regarding the fidelity of the repair, while karyotyping only detects gross chromosomal rearrangements [20,21] and does not detect small deletions/insertions or single nucleotide perturbations. The latter can occur as a result of deficiencies in DNA double strand break (DSB) repair [22,23] or other types of DNA repair processes, which were not investigated in either study. Thus, more research into this area of HSC aging is desperately needed because the deleterious effects of small mutations in individual genes have been demonstrated to play a significant role in the development and progression of myelodysplastic syndrome (MDS) [24,25]. This HSC disease [26,27] is associated with advanced age [28,29] and will become a serious problem in the Western world as its elderly population grows. This review discusses the various lines of evidence that support the notion that DNA repair defects play a role in the etiology of MDS.

## 2. Myelodysplastic Syndrome (MDS) Is a Disease of Genomic Instability

**Figure 1:**
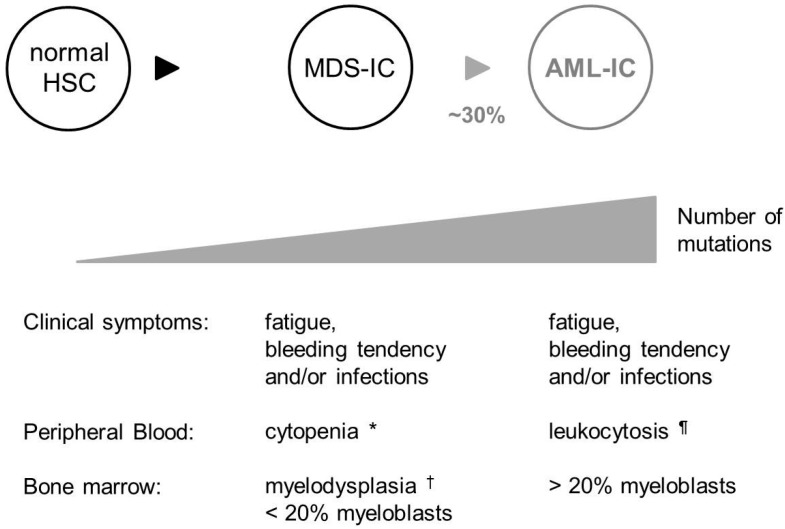
Characteristic features of myelodysplastic syndrome (MDS) in humans. MDS is thought to originate from a mutated Hematopoietic stem cell (HSC). Approximately 30% of MDS patients progress to AML (acute myeloid leukemia) [30]. With each stage, an increased number of mutations in essential genes can be observed [24,31]; the classic representation of both diseases is depicted. Although MDS and AML have very similar clinical symptoms, they can be distinguished from each other by cell counts in the peripheral blood and pathological review of the bone marrow. Classical MDS is associated with myelodysplasia in the bone marrow and cytopenia in one or more myeloid lineages in the peripheral blood. However, a level of >20% myeloblasts in the bone marrow is indicative for AML and excludes the diagnosis of MDS [39,40]. Signs of myelodysplasia can still be present in bone marrow samples of MDS patients that have progressed to AML. In some patients, myelodysplasia and <20% myeloblasts in the bone marrow is accompanied with cytosis in one of the peripheral blood lineages; these patients are diagnosed with MDS/MPN (myleoproliferative neoplasm) overlap disease, which is a different disease altogether [41]. MDS-IC, MDS initiating cell; AML-IC, AML initiating cell; * Cytopenia, significantly fewer cells than normal in one of the peripheral blood lineages; ^¶^ Leukocytosis, significantly more leukocytes than normal in the peripheral blood; **^†^** Myelodysplasia, abnormal blood cell morphology.

MDS patients present with signs of a degenerating hematopoietic tissue: chronic fatigue (due to a lack of functional red cells), unexplainable bleedings and bruises (lack of platelets) and/or recurrent infections (lack of leukocytes). MDS is furthermore characterized by genomic instability and a high propensity to progress into acute myeloid leukemia (AML (acute myeloid leukemia); Figure 1) [30].

Genomic instability is a condition in which cells are prone to acquire and accumulate permanent genomic alterations. Examples in MDS patients include the presence of increased numbers of micronuclei in lymphocytes compared to age-matched healthy individuals [31]. Moreover, there is a direct correlation between the frequency of micronuclei observed and MDS severity [31]. Another example is the presence of microsatellite instability (MSI) in patients with therapy-related MDS/AML (t-MDS/AML) (See the section “Mismatch Repair” for more details). Most importantly, 74%–90% of MDS patients have mutations in one of ~50 known cancer genes [24,25]. The top two categories of mutated genes encode regulators of RNA splicing machinery (*U2AF35*, *ZRSR2*, *SRSF2* and *SF3B1*) and DNA methylation (*TET2*, *DNMT3A*, and *IDH1/IDH2*). Other important categories of mutated genes include: transcription factors and other transcriptional regulators such as *RUNX1*, *MECOM* and *CEBPA*, and chromatin remodeling proteins such as *ASXL1*, *EZH2*, *ATRX*, *KDM6A*, *CREBBP* and *EP300*. Proteins implicated in tyrosine kinase-associated pathways such as FLT3, CBL, RAS, KIT, CSF1R, PTPN11, as well as JAK2 and MPL (both of which are less common and are often acquired during disease progression) and those that regulate cell cycle and apoptosis, including TP53, IER3 and NPM1 are also often somatically mutated in MDS genomes (reviewed in [32,33,34]). In addition, cytogenetic abnormalities are found in approximately half of the MDS patients at diagnosis [35]. A considerable proportion of MDS patients present with the same chromosomal abnormalities: 5q−, −7/7q− and +8 are found in 5%–10% of *de novo* MDS [35] and in 40%–50% of t-MDS patients [36], whereas uniparental disomy at 4q is found in 8% of MDS patients [37]. Less frequent chromosomal abnormalities include −18/18q−, 20q−, −5, −Y, t(17p), +21, inv/t(3q), −13/13q− (present in 3%–5% of MDS patients), and −21, t(5q), +11, del(12p), del(11q) and t(7q) (in <1% of patients [32]). In contrast to AML, balanced chromosomal translocations are rarely found in MDS, the most frequently occurring of these, inv(3)(q21q26.2) or t(3;3)(q21;q26.2), is present in approximately 1% of patients [38].

Importantly, within the same patient, the number of DNA perturbations (mutations and or other DNA abnormalities) often increases as the disease progresses [24,31]. With each additionally required mutation, the risk of leukemic progression, which is associated with a poor prognosis, increases [24].

## 3. MDS Is Associated with Excessive DNA Damage

Genomic instability is directly associated with an inability to cope with damaged DNA, either because of deficient response/repair mechanisms or because of too much DNA damage. Under normal conditions, our genome is constantly exposed to genotoxic stress, either through the formation of genotoxins within the cell (such as reactive oxygen species (ROS) causing oxidative damage), or from the external environment. It is estimated that on average approximately 800 DNA lesions occur per cell per hour [42]. These lesions will be repaired by one of the many DNA repair processes or, when the damage is too extensive, cells will be eliminated. When these coping mechanisms are failing, DNA damage beyond normal levels and permanent DNA perturbations become evident.

In MDS patients, excessive DNA damage is commonly observed in the form of oxidative DNA damage. One study showed significantly elevated levels of oxidized nucleotides in bone marrow cells obtained from MDS patients compared to that of healthy controls (9.2%* vs.* 1.7%) [43]. In another, oxidative damage was measured using fluorescence activated cell sorting (FACS) to quantify levels of 8-oxoguanine (8-OG), one of the most common DNA lesions induced by ROS [44]; two thirds of MDS patients had higher 8-OG levels within their leukocytes compared to healthy controls [45]. Using the same methodology, Peddie and colleagues have shown that this damage could only be found within the CD34+ bone marrow cell population from MDS patients (which includes the HSC population), but not in more differentiated CD34− cells, nor in healthy donor marrow cells [46].

In addition to intracellular sources, the external environment also introduces damage into our DNA. Two of the many environmental genotoxins, γ-radiation and benzene, are well-documented agents that promote the subsequent development of MDS. γ-Radiation is a high frequency electromagnetic wave produced by the decay of high-energy states in atomic nuclei and can cause many forms of DNA damage through the generation of ROS and DNA DSBs. It is naturally found in cosmic rays; this presents little problem for the general human population since it is largely blocked by the Earth’s atmosphere. Yet, radiation poisoning is a concern for astronauts and frequent fliers at high altitudes [47]. People are also at risk if they live near nuclear disasters such as those that occurred in Chernobyl (Ukraine) or in Hiroshima and Nagasaki (Japan). Investigation of clean-up workers from the Chernobyl accident and survivors of the Hiroshima/Nagasaki bombing suggests that radiation poisoning contributed to MDS [48,49,50,51].

Benzene (C_6_H_6_) is another environmental contaminant that causes DNA damage [52] and for which exposure has been associated with an increase incidence of MDS/AML [53]. It is a colorless volatile liquid hydrocarbon found in coal tar and petroleum and used to make numerous chemical products including detergents, insecticides and motor fuels. Benzene is metabolized to benzene oxide in the liver and then further metabolized to phenol, catechol, hydroquinone and 1,2,4-trihydroxybenzene. These metabolites enter the blood and undergo peroxidase-mediated metabolism in the bone marrow [52], where they are converted into several metabolites, including *p*-benzoquinone, the primary causal agent of myelotoxicity. *p*-Benzoquinone binds to proteins and DNA, where a variety of DNA damage is formed, including DNA DSBs [52,54] and also inhibits DNA topoisomerase II, leading to more DSBs. These were shown to cause illegitimate non-homologous end joining (NHEJ)-mediated recombination at a known breakpoint cluster region of the human *AML1* gene [55].

The second largest group of people (after the elderly) that are at risk of developing MDS, are cancer survivors, especially when they have been treated with alkylating agents (such as temozolomide and cyclophosphamide), alone or in combination with topoisomerase II inhibitors (e.g., doxorubicin and etoposide) [56,57,58,59,60,61] and/or radiation (recently reviewed in [62]). Alkylating agents attach an alkyl group (C*_n_*H_2*n*+1_) to DNA, which can be mutagenic or render DNA incapable of properly unwinding for transcription or duplication. The result is cessation of cell growth and apoptosis. However, in some cases, lesions generated by an alkylating agent can be repaired through the combined efforts of various DNA repair pathways (for example, see [63]). Unfortunately, repair of such lesions does not always occur accurately, which can lead to permanent and potentially cancer (MDS)-promoting DNA mutations.

Topoisomerase II cleaves both strands of DNA to relieve supercoiling and tangling. Etoposide, a topoisomerase II poison, covalently binds topoisomerase II to the enzyme-induced DSBs resulting in a ternary drug-enzyme-DNA complex that blocks the replication fork [64]; thus, etoposide converts topoisomerase II into a poison. This type of damage is different from that caused by γ-radiation, which simply breaks DNA strands [65]. Although it remains unclear how these ternary drug-enzyme-DNA complexes are identified and processed, etoposide appears to up-regulate NHEJ and p53-dependent cell killing [66].

Taken together, these studies show that both intracellular and extracellular genotoxins are known to cause DNA damage and some of these genotoxins have been proven to play an important role in promoting MDS development.

## 4. Cellular Mechanisms to Protect against Permanent DNA Damage

**Figure 2:**
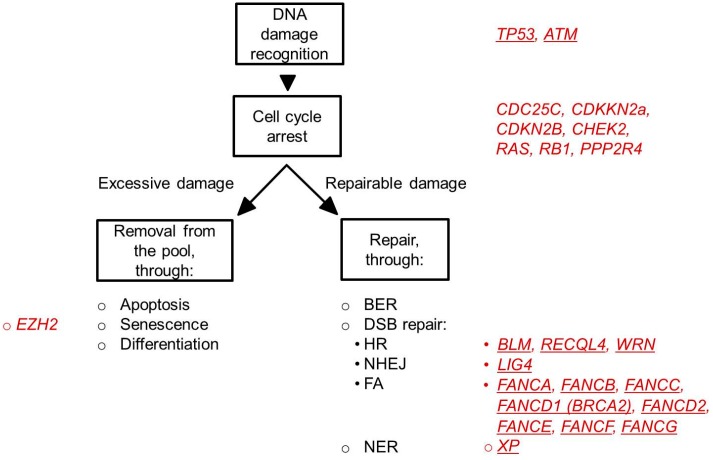
Cellular response to DNA damage. Depicted are the major cellular processes (in black letters) that are initiated after DNA damage. In red are the genes indicated that are related to these processes and that have been found mutated in MDS patients. The underlined symbols indicate genes that are perturbed in hereditary syndromes associated with a high risk of developing MDS (see also Table 1). MDS-associated perturbations in TP53 can be both inherited (Li-Fraumeni syndrome) as well as acquired.

To protect against permanent DNA damage and its potentially deleterious effects, cells are armed with a plethora of defense mechanisms, including DNA damage sensing mechanisms, cell cycle arrest, apoptosis, senescence and differentiation and DNA repair (Figure 2). Which of these are used depends on the nature and the amount of damage, the cell type and stage of the cell cycle in which the damage occurred [67,68]. Failure to properly execute these processes, may lead to the propagation of cells containing genomic abnormalities. Below follows a more detailed description of the various processes utilized by cells in response to DNA damage. Evidence for a possible link to MDS in humans will be discussed.

### 4.1. DNA Damage Checkpoints

To ensure the propagation of cells without genomic aberrations, three major DNA damage checkpoints exist in mammalian cells: the G1/S checkpoint that prevents replication of damaged DNA, the S-phase checkpoint that assesses whether the entire genome has been faithfully replicated before cell division, and the G2/M checkpoint that prevents propagation of damaged chromosomes into daughter cells through mitosis [69,70,71]. Activation of one of these checkpoints delays cell cycle progression, allowing repair of the damage through utilization of a variety of damage-specific processes (see below). Genetic or epigenetic alterations of genes involved in these processes, including *TP53* [72], *RAS* [73], *CDC25C* [74,75], *PPP2R4* [74,75], *CDKN2B* (*p15INK4B*) [76,77], *CDKN2A* (*p16INK4A*) [77], *CHEK2* [78] and *RB1* [79] have been identified in patients with MDS; they are associated with advanced stages of MDS and leukemic transformation. Patients with mutations in TP53 have a significantly worse prognosis than patients without this mutation [80,81,82,83,84,85].

If the DNA damage is too extensive, apoptosis, senescence or differentiation will be initiated [86,87,88] in order that these cells will be purged from the tissue. Several studies have demonstrated that especially in the early stages of MDS, apoptosis is markedly increased. Pro-apoptotic regulators, such as *BID* and *BAX* are upregulated in the red cell precursors of MDS patients [89], whereas expression levels of *IER3* and* BCL-2*, two anti-apoptotic genes, are decreased [90,91,92]. Interestingly, apoptosis is an important mechanism utilized by human HSCs to cope with DNA damage [93]. Although apoptosis has not been measured in the MDS-HSCs itself, it is possible that genomically-compromised HSCs are removed from the pool at this phase of the disease. In advanced stages of MDS (with progression to AML, which is associated with a poor prognosis), expression levels of anti-apoptotic genes are increased and the disease reflects a stage of proliferation rather than cell death [90,94]. Cellular senescence is another strategy through which cells can be eliminated from the HSC pool. One of the genes orchestrating this process is *EZH2* (enhancer of zeste homolog 2). *EZH2* encodes a histone methyltransferase and is a member of the Polycomb repressive complex 2 that has been shown to catalyze trimethylation of H3K27 (H3K27me3) leading to transcriptional repression. Decreased H3K27me3 is associated with cellular senescence [95]. Although senescence as a HSC-elimination strategy has not yet been demonstrated in humans, gain-of-function mutations of *EZH2* are commonly found in MDS and are also associated with a poor prognosis [96,97].

### 4.2. DNA Repair Pathways

#### 4.2.1. Base Excision Repair (BER)

This process corrects DNA damage limited to a few bases with an intact secondary DNA structure, resulting from exposure to ROS, radiation or alkylating agents [98,99,100]. The first stage of BER involves the recognition of damaged or inappropriate bases, and their removal by DNA glycosylases, to leave an abasic site. Several DNA glycosylases have been identified, some of which are strictly lesion-specific, while others are able to recognize multiple lesions. APEX1 (apurinic/apyrimidinic endonuclease-1) then cleaves these sites, creating a single-strand break. Finally, depending on the number of excised nucleotides, the gap is filled by either short-patch BER, in which a single nucleotide is replaced using DNA polymerase-β and the DNA ligase 3/XRCC1 complex, or by long-patch BER, where 2–10 new nucleotides are inserted using DNA polymerase-δ/ε, PCNA and DNA ligase 1 [101].

BER is the major repair pathway to correct ROS-mediated DNA damage. Although abnormal expression levels of genes essential to this repair process have been found in MDS patients [45], a direct causal relationship between decreased BER activity and MDS remains to be determined.

#### 4.2.2. DNA Double Strand Break Repair

Homologous recombination (HR) and NHEJ are the two major DNA repair pathways in mammalian cells responsible for repairing DSBs that can result from exposure to ROS, radiation and certain types of chemicals [102]. Their utilization is largely dependent upon the phase of the cell cycle in which the damage occurs, and also by the nature of the DNA break. HR mainly functions in late S- or G2-phases of the cell cycle because it requires a homologous chromosome or sister chromatid to serve as the repair template. NHEJ, however, does not need a homologous chromatid template and is therefore active in G1 or G0. [103].

Two major models are used to describe HR: DSB repair and synthesis-dependent strand annealing [104]. These two pathways share the initial steps, namely resection of a DNA DSB so that a 3' single-stranded DNA (ssDNA) overhang becomes available as well as subsequent strand invasion by the 3' ssDNA overhang onto a homologous sequence and DNA synthesis at the invading end. Next, in the DNA DSB repair model, the second DSB end can be captured to form an intermediate with two Holliday junctions. After gap-repair DNA synthesis, using the sister strand as a template and ligation, the structure is resolved at the Holliday junctions in a non-crossover or a crossover mode. In the alternative model, no Holliday junctions are formed and instead, the invading strand is displaced after repair synthesis and reanneals with the single-stranded tail on the other DSB end. Essential proteins for HR include the recombinase RAD51 (strand invasion), BRCA2 (delivers RAD51 to the DNA) and others (extensively reviewed in [104]).

NHEJ is more error-prone than HR due to its ability to process DNA ends prior to ligation, and may generate deletions through removal of nucleotides at damaged ends, or form translocations via ligation of non-matching termini. The classical NHEJ pathway utilizes repair machinery consisting of DNA-PK, XRCC6 (KU70), XRCC5 (KU80), POL IV and DNA ligase 4/XRCC4 complex [105,106]. Depending on whether the single-strand overhangs on the DSB ends are compatible or not, no processing or minimal processing of these ends is performed prior to ligation, leading to accurate or imprecise repair, respectively [105]. There is convincing evidence that the absence or down-regulation of components of the classical NHEJ pathway in leukemia cells induces the activation of an alternative, DNA-PK independent and even more error-prone NHEJ pathway: the microhomology-mediated end joining (MMEJ) pathway [107,108,109]. This resects nucleotides from the broken ends until a complementary sequence (normally 5–25 base pairs in length [110]), or microhomology, is revealed on both strands [111]. Microhomologous sequences are then utilized for aligning the broken strands before joining. As a result, nucleotides between adjacent microhomologies are deleted. This is in contrast with classical NHEJ which either simply joins the ends and results in no deletion or typically uses microhomologies already exposed in overhangs on the DSB ends (up to 5 base pairs) and result in short deletions (1–4 nucleotides).

Recent studies in mice have revealed that classical NHEJ is utilized by quiescent HSCs to maintain their genomic integrity (please see [112] for an extensive discussion of this topic). Subsequent work from De Laval *et al.* showed that thrombopoietin (TPO) signaling, via its receptor (MPL) and downstream activation of ERK and NFKB1, leads to increased levels of phosphorylated IER3. The latter is essential to promote classical, DNA-PK-mediated NHEJ in stem and progenitor cell populations after exposure to ionizing radiation [113,114]. γ-Radiation induces prolonged genomic instability in HSCs and their progeny [115]. De Laval* et al.* furthermore demonstrated that this was more severe in HSCs that lacked MPL, while TPO admission to wild-type HSCs prior to radiation exposure diminished long-term genomic instability. Since MDS is a HSC disease, it is possible that this TPO/DNA-PK-mediated NHEJ repair pathway in HSCs is defective and that, as in leukemias, excessive MMEJ is responsible for the genomic instability observed in MDS patients. Although there is no experimental evidence to support this notion in patients yet, it is interesting to note that various IER3 perturbations have been found in MDS patients [92,116] and that *Ier3* knock-out mice develop a bone marrow failure syndrome with MDS-like features [117].

The Fanconi anemia (FA) pathway interacts with both the HR and NHEJ pathways. It is particularly important for correcting DNA lesions that stall a replication fork such as interstrand crosslinks. Sixteen FA genes have been identified which are categorized into three groups based on their function [118,119]. Group 1 proteins, including, FANCA, B, C, E, F, G, L and FANCM, form a core complex that identifies the lesions and serves as E3 ubiquitin ligase, which monoubiquitinates the group 2 proteins (FANCI and FANCD2). Group 3 proteins, including FANCN, FANCD1 (also known as BRCA2) and FANCJ, mediate crosstalk with other DNA repair pathways including HR and NHEJ [120,121].

Chromosomal deletions and translocations are typical outcomes of aberrantly repaired DSBs. The former is frequently found in MDS, while the latter occurs in t-MDS but rarely in *de novo* MDS. Mouse studies have demonstrated that misrepair through aberrant NHEJ can also lead to point mutations and small deletions/insertions [23], which would fit with the mutation spectrum found in MDS patients. Indeed, patients with genetic disorders resulting from perturbations in DNA DSB repair genes have an increased incidence of MDS development (Table 1). Most prevalent among these disorders is Fanconi anemia, which is a congenital bone marrow failure syndrome with a hypersensitivity to genotoxic agents, such as mitomycin-C, that create replication fork-blocking lesions. Approximately 20% of the Fanconi anemia patients develop MDS [122] with a genomic instability that progresses with each state of the disease [123]. Other DSB repair deficiency syndromes with a high incidence of MDS are patients with a defect in a RecQ helicase such as Bloom syndrome [124], Rothmund-Thomson syndrome [125,126,127,128] and Werner syndrome [129,130].

Acquired perturbations in DSB repair genes or DSB repair activity are not a common finding in MDS patients. This is in contrast to AML, where it is becoming increasingly clear that inefficient DSB repair contributes to disease etiology [131,132,133,134]. Possible explanations for this apparent discrepancy may be that DSBs are more likely to be lethal for the cell, or that the consequences of aberrantly repaired DSB are more serious than other types of repair and will lead directly to leukemia.

**Table 1:** Increased incidence of MDS in pediatric syndromes associated with defective DNA repair.

Process Involved	Disease	Gene(s) Involved *	Incidence of Disease	Incidence of MDS in Patients ^§^
DNA damage response and cell cycle checkpoint	Li-Fraumeni syndrome [135]	*TP53* or *CHEK2*	400 Cases reported	3 Cases reported
**DSB repair**				
Global	Ataxia Telangiectasia [136]	*ATM*	1/100,000 to 40,000	Not reported
HR	Bloom syndrome [124]	*BLM*, *BLAP75*/*RMI1*	1/48,000	4 Cases reported
	Rothmund-Thomson syndrome [125,126,127,128]	*RECQL4*	300 Cases reported	4 Cases reported
	Werner syndrome [129,130]	*WRN*	1300 Cases reported	6 Cases reported
FA pathway	Fanconi anemia [122]	*FANC*(*A*–*G*)	1/350,000	20.7%
NHEJ	Lig4 syndrome [137]	*LIG4*	Few cases reported	1 Case reported
NER	Xeroderma Pigmentosa [136]	*XP*	1/250,000	Not reported
**Other ^¶^**				
Unclear	Neurofibromatosis type 1 [138]	*NF1*	1/3500	200–500-Fold increase in children
	Shwachman-Diamond syndrome [139,140,141]	*SBDS*	1/50,000	8%–33%
Telomere maintenance	Dyskeratosis congenital [142,143,144]	*DKC1*, *NOP10*, *NHP2*, *TERC*, *TERT*, *TINF2*	1/1,000,000	4%–5%
Oxidative DNA damage repair	Down syndrome [145,146,147]	Trisomy 21	1/1000 to 1/650	1/1000 to 1/500

* Genes involved are the genes thought to be directly responsible for the genetic syndrome mentioned in the corresponding “disease” column; **^§^** Most of these diseases present themselves in early childhood. The overall incidence of *de novo* MDS in children is ~1.8–4 per 1,000,000 [148]; ^¶^ Although these diseases are not considered classical DNA repair-deficiency disorders, patients suffering from them have shown either a compromised DNA damage response or increased potential to acquire DNAlesions [139,146,149,150]; Part of this table was published previously in [112].

#### 4.2.3. Mismatch Repair (MMR)

The MMR pathway corrects base-base mismatches and DNA loops, resulting from aberrant DNA deletions or insertions; when a deletion occurs on the nascent strand after replication, the complementary sequences create a loop on the template strand. Conversely, when an insertion occurs on the nascent strand, the inserted sequence forms this secondary structure. Similar to other excision repair pathways, MMR starts with damage recognition and removal, followed by DNA resynthesis and ligation. In mammalian cells, two heterodimeric complexes have been identified that recognize mismatches: hMutSα (MSH2–MSH6) and hMutSβ (MSH2–MSH3), which are recruited to the site of damage by PCNA [151]. The endonuclease hMutLα (MLH1–PMS2) then creates a nick at the error site. Upon DNA unwinding by an as yet unknown helicase, 5'→3' exonuclease 1 (EXO1) excises the damaged site and surrounding nucleotides, creating a single-strand gap. This gap is then repaired by the combined action of PCNA/polymerase-δ complex and DNA ligase I, using the other strand as a template (extensively reviewed in [152]).

Defective MMR in protein coding regions of the genome can result in frameshifts and/or premature stop codons leading to the production of truncated, inactive proteins [153]. Microsatellite regions (*i.e.*, DNA repeats of 1 to 7 base pairs) are non-coding areas of the genome especially prone to replication errors that result in base-base mispairing and DNA loop formation. When MMR functions optimally, the copy number of repeats in such areas is the same in all cells. However, when MMR is impaired, cells begin to display variable numbers of repeats for a given locus, creating so-called microsatellite instability (MSI) regions. MSI regions could be demonstrated in 14%–64% of t-MDS/AML patients [154,155,156], suggesting that MMR deficiency in these patients is a common occurrence. Interestingly, mouse studies showed that MMR-deficient cells have a survival advantage over wild-type cells when exposed to temozolomide [157], a methylating agent used to treat certain brain tumors and known to be associated with an increased risk for t-MDS/AML development [157,158,159]. Thus, one possible mechanism through which t-MDS/AML develops is selective survival of MMR-deficient cells, allowing those carrying potentially harmful DNA mutations in protein coding regions to propagate.

#### 4.2.4. Nucleotide Excision Repair (NER)

The NER pathway, consisting of global genomic repair and transcription-coupled repair [158], removes DNA lesions that affect proper DNA double helix formation (for example, UV-induced thymine dimers and pyrimidine (6–4)-pyrimidone photoproducts, and carcinogen-induced adducts and cross-links [159]). These two pathways differ essentially only in the proteins used to recognize damage [160], namely XPC (xeroderma pigmentosum, complementation group C)-hHR23B-centrin-2 protein complexes [161] and RNA polymerases [162,163], respectively. Following damage recognition, the excision and resynthesis steps appear to be shared by both pathways. ERCC3 (excision repair cross-complementation group 3) and ERCC2 helicases open the DNA structure around damage sites, then ERCC5 and ERCC4-ERCC1 nucleases remove DNA surrounding the sites of damage, leaving a DNA gap of 24–32 nucleotides. This is filled, using the other strand as a template, by DNA Polymerase-δ or -ε with the help of proliferating cell nuclear antigen (PCNA), and sealed by DNA ligase (reviewed in [164]).

Hereditary defects in genes encoding essential NER proteins result in various diseases, including xeroderma pigmentosum (XP), Cockayne syndrome and trichothiodystrophy [165,166]. XP (but not the others) is associated with an increased risk of MDS [167,168] (Table 1). Moreover, treatment with Fludarabine, a chemotherapeutic drug that inhibits NER activity, is associated with an increased occurrence of t-MDS/t-AML [169,170].

## 5. Conclusions

The following observations all point to MDS being a disease associated with an inability to adequately respond to DNA damage: (1) Genomic instability is an essential feature of MDS, which worsens as the disease progresses; (2) Hereditary syndromes caused by, or associated with, abnormal DNA repair show a significantly higher risk for MDS development (Table 1); and (3) Exposure to excessive amounts of genotoxins, including chemotherapeutic agents, causes MDS. Yet, aberrations in DNA damage response/repair genes, other than TP53 and some genes involved in DNA damage checkpoints (Figure 2), are rarely found in adult MDS patients. However, MDS is also considered a classical “epigenetic disease” where DNA methylation, histone modification and microRNA expression can be affected (reviewed in [171,172]). Epigenetic changes affect cellular function and this may include DNA repair, although, this has not been investigated yet. Furthermore, many MDS patients have been shown to acquire mutations in genes with a role in epigenesis, including *TET2*, *IDH1*, *IDH2*, *ASXL1*, *EZH2* and *DNMT3A* (as reviewed in [173]). These, however, are not specific to patients with MDS, but are commonly seen in other myeloid malignancies.

Recent experimentation into the relationship between the dose of a genotoxic agent and its mutagenic potential suggest that at low doses, normal cellular processes (DNA repair) are able to fix the damaged DNA; mutagenicity seem to occur only if the damage is beyond a certain threshold [174]. Whether this phenomenon is true for all genotoxins remains to be determined; However, the notion of a threshold effect is of potential interest in light of understanding MDS susceptibility. It is tempting to speculate that a DNA damage threshold exists for each tissue, and that this is specific to each person, depending on their genetic make-up. Gene polymorphisms are likely important determinants of these thresholds; those that significantly lower the threshold in hematopoietic cells are thus expected to be associated with a higher susceptibility to MDS and other hematopoietic malignancies. Aging, with a normally occurring alteration in the epigenetic signature of cells, including HSCs [175], may effectively lower the DNA damage threshold even further. This scenario is not without support. In an excellent review by Guillem and Tormo, many examples are provided that link certain polymorphisms of DNA repair genes to increased risk of developing MDS/AML after chemotherapy [176]. Another example includes the BER-related gene *OGG1*. A naturally occurring polymorphism of this gene (*OGG1-Cys326*) was found more often in MDS patients compared to controls [45]. Research into the exact nature of the relationship between gene polymorphisms, DNA damage response/repair and cancer risk is an emerging field, but it may reveal important biomarkers for people at high risk to develop specific age-related cancers such as MDS or MDS secondary to chemotherapy or other genotoxic exposures.
